# Constitutions Ruined

**Published:** 1857-05

**Authors:** 


### CONSTITUTIONS RUINED.

IT is natural enough that a poor youth taken from manual labor in high health, then sent to engage in studies preparatory to professional life, should, after years of alternate eating, sleeping, and study, fall into disease, and die of consumption, just as he is finishing his studies! A few survive a little longer, enter their profession, and in a year or two pass away in death. Among the rarest of instances is it, that professional life is attended with vigorous health.

The Boston *Puritan Recorder* says of one: "He was a young man of great promise, second to no one in his class in scholarship, and had uniformly exhibited a depth of piety and a fixedness of purpose very remarkable. Being indigent, he was assisted by the *American Education Society*, and in every respect merited its support. Cut off, just as he was about to leave college, by a pulmonary complaint." This is not a solitary case. It is one among scores, if not hundreds. There are questions of common sense and of common honesty which we wish to propose in connection with this case, and which we do not intend shall remain unanswered.

The funds of the *American Education Society* are supplied by the voluntary charitable contributions, in large part, of many who work for a living, and in good faith that they will be used judiciously, and in the best direction.

Is the Society as careful to inquire into the bodily health and constitution of the young men who are proposed for their benefactions as a recruiting officer or an insurance clerk? Have they any right to expend the money placed in their hands on young men of doubtful health, under the vague hope of their outgrowing their ailments? They have not.

But suppose the health of the applicant is unexceptionable, have they a right to support him in his studies without any scientific inquiries as to his health subsequently, and without any personal supervision as to that point? They have not. We know from long residence in Louisiana, that the health of a newly-purchased slave is an object of special solicitude on the part of the owner, and continues to be. The health of his men is the first care of a good general or of a veteran commodore. And yet we will venture to guess, that not one dollar, not one hour of attention by salaried employees is expended in the direction of the preservation and promotion of the health of a single beneficiary. No lectures given, no books or periodicals of general hygiene, no personal inquiries, injunctions and requisitions made! If this is not culpable negligence, if this is n:t a shirking of the spirit of the office, the duties of which they are paid to perform, what is it?

This young man studied until he died. What were the professors of Dartmouth College doing all this time? Where was the Secretary of the American Education Society for the three, six, twelve months, of young Hardy's gradual march to the grave? Or, did he have good health until within three months of death, then fall into consumption and die apace? Such could not have been the case. It never is the case. Common consumption of the lungs is the work of years. It always is the work of years. It never is the work of weeks or months.

The *Puritan's* good-hearted correspondent ejaculates, "may the Lord raise up more such who shall be permitted to enter his vineyard with equal promise of talent and piety." Now, this, to our mind, borders on the ridiculous, only we would not treat religious things with unweening levity. Yet we must say, that we see no evidence that the Lord had any hand in preventing this young man's entering the ministry. He prevented himself. He ate as much as he wanted, he slept as much as he wanted, studied hard, and he died, as a very natural result. The faculty at Dartmouth College are salaried men. If all the students die, their salary goes on. Besides, they are not expected to be the mothers, nurses, and physicians of the young men under their charge—the business for which they are paid is to make chalk-marks on the black-board, and talk about themes, roots, derivations, the squares of the distances—*a* plus *b* minus *c* equals *c* minus *b* plus *a*, *quod erat demonstrandum.*

As to the Executive Committee of the Society, their business is to figure out the ways and means, and the Secretary is employed to collect facts, make statements, and publish money-bringing anecdotes. So, after all, these persons are not blamed by us, perhaps, for anything more than one of the grossest oversights of which sensible people can be guilty, and we close by saying that the inattention which prevails in reference to the health of the students in our seminaries, colleges, and universities, is a living disgrace to every teacher and trustee connected with them. And as far as beneficiaries are concerned, it is a gross misuse of the mite of the widow and the penny of the poor, to expend hundreds and thousands of dollars in bringing forward indigent young men into the ministry without personal teachings addressed to each, individually, by competent medical men, from the very first day of their entrance on student life.

